# A novel gelatinized barium sulfate injection method for assessment of bronchoalveolar lavage parameters

**DOI:** 10.1111/crj.13721

**Published:** 2024-01-11

**Authors:** Alexander R. Gross, Temitope Kehinde, Lindsey Morais, Marshall Hutchison, Joy Grise, Nada Mohamed, Varun Badami, Haroon Ahmed, Matthew J. Zdilla, Jeffrey A. Vos, Austin G. Gross, Rachel Leonard

**Affiliations:** ^1^ Department of Pathology, Anatomy, and Laboratory Medicine (PALM) West Virginia University School of Medicine Morgantown West Virginia USA; ^2^ West Liberty University Wheeling West Virginia USA; ^3^ Department of Radiology West Virginia University School of Medicine Morgantown West Virginia USA; ^4^ Department of Pulmonary and Critical Care Medicine West Virginia University School of Medicine Morgantown West Virginia USA

**Keywords:** bronchoalveolar lavage, bronchoscopy, pulmonary anatomy, research

## Abstract

**Introduction:**

Bronchoalveolar lavage (BAL) is frequently used in pulmonary medicine though it requires further optimization. Practical obstacles such as patient safety and procedural limitation have to date precluded large, controlled trials aimed at standardization of BAL procedure. Indeed, BAL guidelines are based on observational data. Innovative research methods are necessary to advance the clinical practice of BAL.

**Methods:**

In our study, we evaluated the effect of injecting a gelatinized barium solution into different lobes and segments of cadaveric lungs. As the technique requires an irreversible injection into lung airspaces, it is not suitable for in vivo purposes. We measured the volume returned from BAL as well as the distribution of BAL injection via dissection. Segmental anatomic orientation was compared to a radiologist's impression of plain film radiographs taken of injected lungs.

**Results:**

Mean injected volume distributions were greatest in the upper lobes and lowest in the lower lobes; mean ratios of injected volume distribution to lung lobe volume also followed this trend. Cannulated bronchi orders favored lower branches in the upper lobe and higher branches in the lower lobes. Segmental anatomy varied by the lung lobe injected and was most varied in the lower lobes.

**Conclusion:**

This novel gelatinized‐barium injection technique provides a minimally complex method to yield clinically meaningful feedback on the performance of BAL. The technique is also adaptable to study of procedural parameters in the context of variable lung anatomies and pathologies.

## Graphical abstract

Cadaveric lung injection with barium gelatin creates a research model for bronchoalveolar lavage that is unachievable clinically. This method can yield new insights and refinements through a combination of clinical laboratory assays, histology, and anatomic dissections. This method can be applied to generate procedural feedback and also study diseases, which are historically challenging to diagnose.

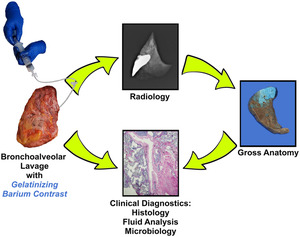

## 1 INTRODUCTION

Bronchoalveolar lavage (BAL) is a minimally invasive procedure, utilized in both diagnostic and research settings, which requires further optimization. Bronchoscopy is often used in clinical practice to evaluate pathology in a predetermined lung lobe. The procedure yields a liquid sample to then submit for select laboratory analyses. Unfortunately, there are no large, controlled trials related to the standardization of BAL procedure; processing of the sample and most recommendations are based solely on observational data.^1^ Significant controversy surrounds semi‐quantitative and quantitative cut‐offs used to identify lower respiratory tract pathogens—PCR and culture‐based methods both utilize the same threshold.^2^, ^3^ These scenarios illustrate several potential opportunities for improvement related to the clinical utility of BAL. One such area is the under‐investigated pathologic characterization of a BAL‐targeted segment of lung.^4^

The quality of BAL samples is defined by the degree that instilled fluid washes alveoli and then is returned from a diseased bronchoalveolar segment. However, characterization of the precise anatomy of instilled fluid relative to its intended radiologic and bronchoscopic target is often preempted by procedural obstacles and patient safety concerns. Also, BAL research study design is confounded by BAL performance that varies by lung lobe. In particular, standardized studies are often complicated by anatomic variability, namely, the irreproducibility of subsegmental bronchial branching as it relates to segmental parenchyma—especially in the larger lower lobes.^5^

To investigate these frontiers of BAL practice and research, we developed a novel method using an injected barium sulfate solution. This method will yield more precise correlation between an intended BAL target and its associated radiologic and clinical laboratory results.

## 2 MATERIALS AND METHODS

Cadaveric studies were performed in the Department of Pathology, Anatomy and Laboratory Medicine morgue at West Virginia University. From 10 cadavers, 19 fresh lungs were explanted and utilized in this study. Twenty lungs were expected although one cadaver underwent pneumonectomy while living, unknown to us; hence, 19 lungs were obtained. Four lung lobes failed to be injected and were excluded from the study. Cadavers ranged from 40 to 96 years of age, 44–113 kg, and included six males and four females. This method requires an irreversible injection to the lung airspace and thus is unsuitable for in vivo applications.

### 2.1 Lavage fluid preparation

We began by defining the total volume of aliquots required. For 20 lungs, 2.0 L of stock solution was mixed (40 mL aliquot × 5 lobes/subject × 10 subjects = 2000 mL). The stock solution was prepared: distilled water (diluent), gelatin powder (0.02 g/mL), barium sulfate powder (0.6 g/mL), and acacia gum powder (0.01 g/mL).^6^ Instead of barium sulfate and acacia, we used the commercially available 98% weight/weight barium sulfate for oral suspension. We added two to three drops of surgical tissue ink per 50 mL of stock solution for contrast with lung tissue.

We dissolved the gelatin powder in hot, distilled water in a securable vessel then added oral contrast solid. Surgical ink was added at this time and the solution shaken well to mix—then stored at room temperature.

### 2.2 Lung injection

We started by explanting the lungs with standard autopsy methods. Surgical ink was applied to the pleura on each lobe to designate anterior, posterior, and mediastinal surfaces and the lateral aspect.

We prepared a mini‐BAL catheter (Ballard‐Avanos, Alpharetta, GA) and a wash basin filled with hot tap water. Our lungs specimens were refrigerated, which hastens gelatinization.

First, we incubated the stock solution in hot tap water until contents were liquefied. We then navigated the cannula to the intended bronchus until wedged (Figure S1). The stock solution was agitated to disperse the solids, drawn up into the desired aliquot volume with a Luer‐Lok syringe (BD, Franklin Lakes, NJ) and injected 3–5 mL/s and the cannula left in place—1 min with cold specimens, 3 min at room temperature. Finally, the lungs were inflated fully with 10% formalin using standard post‐mortem techniques and stored at room temperature.

### 2.3 Imaging

**FIGURE 1:**
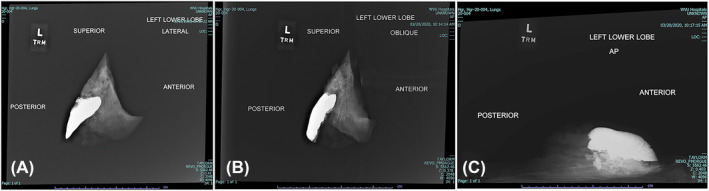
Left lower lobe, plain X‐ray—(A) lateral, (B) oblique, and (C) anterior–posterior view. Careful positioning allows for correlation of radiologic images and gross photos.

We obtained plain film radiographs of explanted, individual lung lobes after injection (Figure 1). At the time of imaging, we separated the lobes by transecting the lobar bronchi with a trimming blade, noting anatomic variations. We established, with a radiology technician, a reproducible and consistent orientation of the intact lobes and performed anterior–posterior, lateral, and oblique radiographs. Surgical towels and foam shapes assisted in positioning the lungs for radiography; inked pleural landmarks applied to lungs prior to explant also aided in orientation. Each lobe was stored individually in labeled containers after imaging.

### 2.4 Dissection

We worked lobe‐by‐lobe. A metal probe was inserted through the lobar bronchus towards the site of injected lung. Injection fluid was visible through the pleura, indicating where to direct the probe. Then, beginning at the pleura furthest the probe tip, the injected lung was sectioned in approximately 1.0 cm slices, perpendicular to the probe. When the sections revealed the bronchus where the injection was made, the bronchi was dissected along the probe from proximal to distal. The pertinent bronchial order branching and anatomical course was noted. Lastly, the probe was removed, and the remaining uninvolved lung sectioned into 1 cm slices.

**FIGURE 2:**
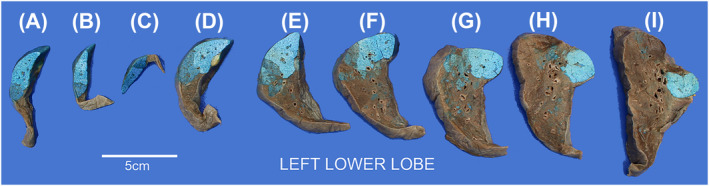
Left lower lobe post‐injection, gross specimen. Labeled 1.0 cm thick slices allow for precise volume measurement and segmental assessment of BAL injection.

At this point, we facilitated volume measurements with digital image analysis software (i.e., ImageJ, NIH, Bethesda, MD) by (1) arranging and labeling each slice with letters A, B, C, so forth, (2) measuring and noting down slice widths by their corresponding letter, (3) taking a digital photo of the slices, and (4) outlining the lung involved by gelatinized fluid and the uninvolved lung in ImageJ. Volume was calculated then by thickness of slice × area of injection (or slice) (Figure 2).

**FIGURE 3:**
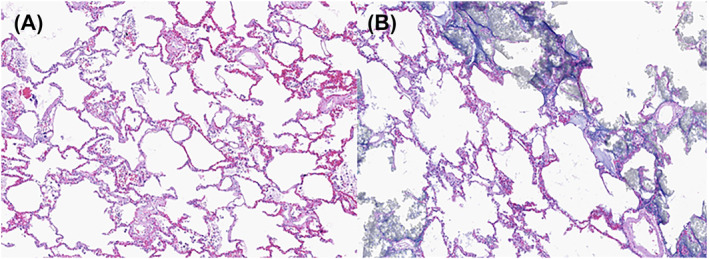
Histology reveals alveolar enlargement characteristic of mild emphysema (A), which is still visible with minimal artifact in the injected lung segment (B). Hematoxylin and eosin, 20×.

Finally, directed by gross or radiologic findings, histologic sections were collected within the injected lung and/or surrounding parenchyma (Figure 3). The anatomic location of sections was noted in a cassette key.

## 3 RESULTS

Anatomic and volume analyses in each case were made based on radiologic images, dissection notes, and digital images as exemplified by Figures 1, 2, and 3.

**FIGURE 4:**
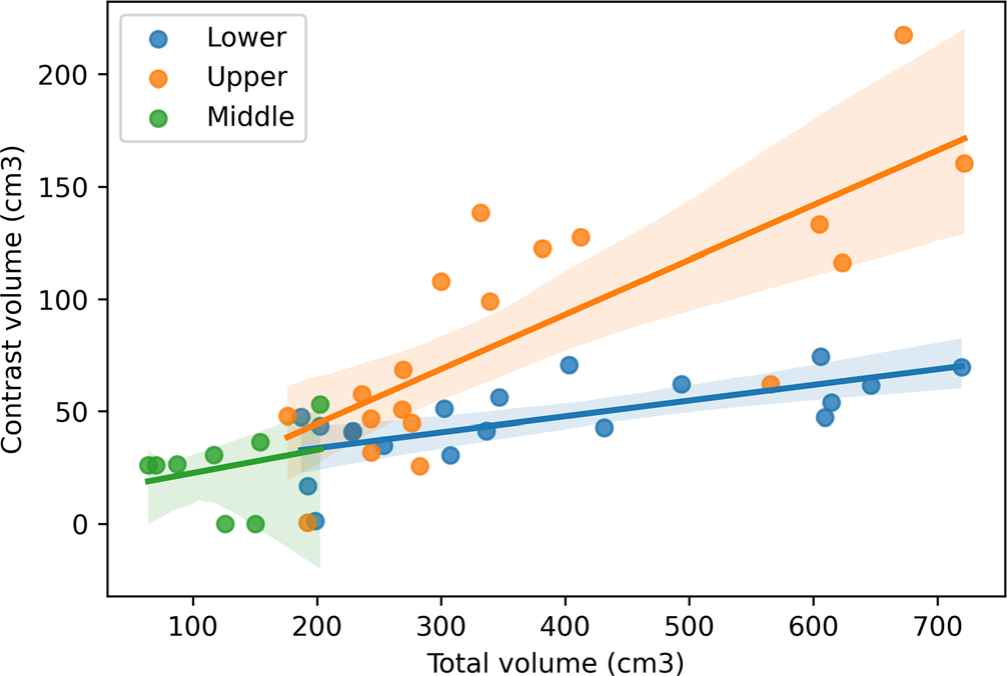
Lung lobe volume versus corresponding distribution of BAL volume.

Among the remaining 10 subjects, measured lung volumes ranged as follows: right upper lobe 391–1635 cc (mean 902 cc), right middle lobe 176–828 cc (mean 401 cc), right lower lobe 648–1454 cc (mean 927 cc), left upper lobe 386–1617 cc (mean 798 cc), and left lower lobe 556–2155 cc (mean 975 cc). The volume distribution of the injected aliquots ranged as follows: right upper lobe 74–161 cc (mean 129 cc), right middle lobe 61–100 cc (mean 78 cc), right lower lobe 54–130 cc (mean 96 cc), left upper lobe 68–418 cc (mean 148 cc), and left lower lobe 56–130 cc (mean 97 cc). The ratio of injected aliquot distributions to total lobe volume ranged as follows: right upper lobe 0.07–0.2 (mean 0.16), right middle lobe 0.12–0.38 (mean 0.24), right lower lobe 0.06–0.18 (mean 0.09), left upper lobe 0.08–0.26 (mean 0.14), and left lower lobe 0.06–0.21 (mean 0.11) (Table 1). The distribution of the injection volumes with standard error is shown by lung lobe volume in Figure 4. The segmental anatomy designated by gross and radiologic correlation are listed in Table 2.

**TABLE 1:** Gross measurements of lung lobe and injection distributions.

Lung lobe	Lobe volume—range, mean (cc)	Injection distribution—range, mean (cc)	Injection distribution:lobe volume—range, mean
Right upper	391–1635, **902**	74–161, **129**	0.07–0.2, **0.16**
Right middle	176–828, **401**	61–100, **78**	0.12–0.38, **0.24**
Right lower	648–1454, **927**	51–130, **96**	0.06–0.18, **0.09**
Left upper	386–1617, **798**	68–418, **148**	0.08–0.26, **0.14**
Left lower	556–2155, **975**	56–130, **97**	0.06–0.21, **0.11**

**TABLE 2:** Bronchoalveolar lavage segmental anatomy by gross and radiologic orientation.

		Subject 1	Subject 2	Subject 3	Subject 4	Subject 5	Subject 6	Subject 7	Subject 8	Subject 9	Subject 10
Right upper lobe	Radiology	Ant/post	Ant	Api‐post	Api	Ant	Api‐post	‐	Ant/sup	Ant/post	Ant/post
	Gross	Post	Ant	Post/Sup	Sup/ant	Post	Ant/sup	‐	Ant/sup	Api‐post	Post
Right middle lobe	Radiology	Med/lat	Med/lat	Med/lat	Lat	Lat	Lat/med	‐	Lat/med	‐	‐
	Gross	Med/lat	Lat	Med/lat	Lat/med	Lat	Lat/med	‐	Lat/med	‐	‐
Right lower lobe	Radiology	Posterior	Lat/med	Med	Ant	Ant	Post	‐	Lat	Post	Lat
	Gross	Posterior	Lat/post	Post/lat	Ant/med	Post	Post	‐	Lat	Ant/med	Post/med
Left upper lobe	Radiology	Api‐post	Ling	‐	Ant	Ant	Api‐post	Api‐post	Inf	Ant	Sup
	Gross	Ant	Ant/inf	‐	Ant/inf	Api‐post	Ant/sup	Api‐post	Inf	Inf	Ant/inf
Left lower lobe	Radiology	Sup/ant	Ant	‐	Lat	Lat	Post	Post	Lat	Post	Sup
	Gross	Ant	Post	‐	Ant/med	Post	Post	Lat	Post	Lat	Sup

Abbreviations: Ant, anterior; Api‐post, apico‐posterior; Inf, inferior; Lat, lateral; Med, medial; Post, posterior; Sup, superior.

**FIGURE 5:**
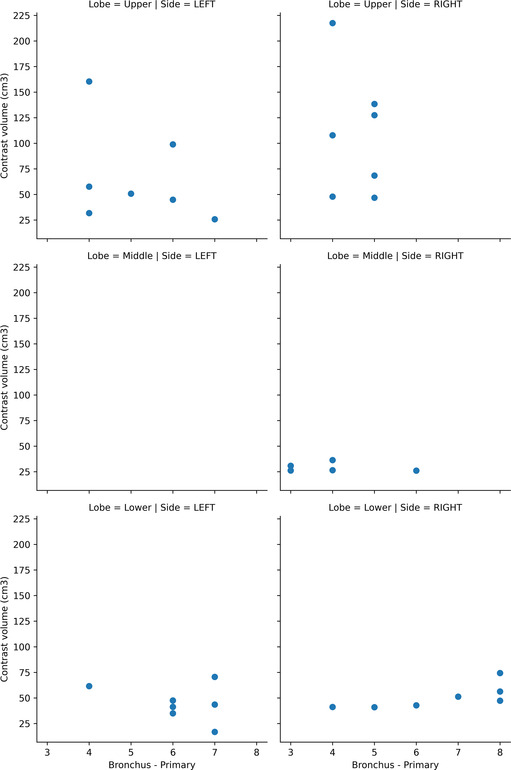
Bronchial order cannulated by long lobe.

The cannulated branch order of bronchi in each lobe is as follows: right upper lobe 4th (*n* = 3) and 5th (*n* = 4); right middle lobe 3rd (*n* = 2), 4th (*n* = 2), and 6th (*n* = 1); right lower lobe 4th (*n* = 1), 5th (*n* = 1), 6th (*n* = 1), 7th (*n* = 1), and 8th (*n* = 3); left upper lobe 4th (*n* = 3), 5th (*n* = 1), 6th (*n* = 2), and 7th (*n* = 1); left lower lobe 4th (*n* = 1), 6th (*n* = 3), and 7th (*n* = 3). The distribution of injections volumes by bronchial order is shown in Figure 5 divided by their lung lobe.

## 4 DISCUSSION

Bronchoscopy is a commonly utilized procedure for invasive sampling of the lungs. Unfortunately, large studies evaluating radiologic and histologic correlation have not been investigated. Prior to planned procedures, imaging is reviewed, and appropriate lobes and subsegments are selected for performing bronchoalveolar lavage. Aliquots of sterile saline are instilled until an adequate return of sample (>5% of instilled fluid) is obtained to send for analysis. Procedural variables and patient safety limits research methods for the BAL procedure.

Various radio‐opaque materials including barium have been used in the past to study airway anatomy. Historically, bronchographic methods were used routinely with different substances including oily Dionosil prior to the advent of Computed Tomography.^7^ This prior technique cannot be used to study distribution of radio opaque material into the alveoli as it does not penetrate beyond the terminal bronchioles.^8^ There is also concern about the safety of this procedure given significant side effects cause from injection of these substances, including hypoxia, bronchospasm, and even death.^9^ Our technique looks at the utility of radio‐opaque material to evaluate distribution of alveolar lavage fluid past the terminal bronchioles and in the alveoli. Digital subtraction radiography has been used in vivo, but prior attempts were limited to 2D radiography without gross anatomic or histopathologic correlation.^10^

Our novel method allows for evaluation of more precise anatomical distribution when performing BAL with clinical, radiologic, cytopathologic, and microbiological correlates as required. Of note, although not performed in our study, pre‐injection radiology offers the proceduralist the opportunity to compare their intended radiologic target to their injected lung segment. Other studies have been evaluating this as well. For example, recently several investigators demonstrated that electrical impedance tomography could be used clinically to identify an area of lung where saline is instilled.^11^, ^12^ Previously, a group visualized BAL instilled radio‐opaque fluid using digital subtraction radiography; observations of instilled volume relative to lung anatomy sampled were also recorded.^13^ A handicap of that study was limited imaging resolution; moreover, these studies failed to establish definitive evidence of disease where the BAL was performed. Our method allows a direct correlation between the expected area sampled by BAL (defined by radiology and the proceduralist) and a defined, precisely measured volume of a lobe accompanied by histopathologic mapping of disease.

In our study, the cannula was advanced until its diameter was equivalent to lower order bronchi within the upper lobes and higher order bronchi within lower lobes. This distinction is most evident in the upper lobes when comparing the right and left side. Bronchial diameter decreases quickly in upper lobes and more gradually in lower lobes, partially accounting for the bias noted in larger injection distributions in lower order bronchi of the upper lobes. For example, fluid travels less distance to terminal bronchioles/alveoli in upper lobes and, therefore, larger tissue beds are sampled (vis‐à‐vis lower lobes). Identical injection volumes in the lower lobes leave an unspecified portion of the target segment unsampled. This may be due to inadequate injection volumes or cannulating a bronchus within a minor bronchoalveolar subsegment.

In clinical practice, return of BAL fluid is greater in the upper lobes compared to the lower lobes; a phenomenon resulting from gravity and hydrostatic pressure assisting return in the upper lobes. Also, sensitivity tends to be greater in the upper lobes.^14^ It is critical to instill sufficient volume to adequately inflate a targeted lung segment for sampling; this volume varies by lung lobe. Our findings suggest that the lower lobes' injection volumes should exceed those of the upper and middle lobes particularly if the cannulated bronchus is of lower order. Further work may test if this approach improves sampling and sensitivity of the BAL.

Our study has several limitations. Our data is only from one center; however, we followed rigorous steps for BAL and dissection, which have been included in order to standardize the data. Data collected may not be extrapolated to living lungs given changes in sedation, positioning, and spontaneous or mechanical ventilation. However, our technique could be modified to explore the relationship of position of the lung on the distribution of the gelatinized barium. For example, lungs could be injected in situ in variably positioned cadavers (Figure S2); a fiberoptic bronchoscope could also be utilized instead of blind BAL. Additional tools such as fluoroscopy could be used in conjunction with fiberoptic visualization of airway tree to get more reliable targeting of lung segments. Decreased yield of BAL is also anticipated when patients are under general anesthesia or on mechanical ventilation compared to utilizing local anesthesia.^15^ Our procedure is adaptable by allowing comparison of differences in BAL distribution with different modes of cadaveric ventilation/position to determine the mode that would produce the optimal yield and distribution during BAL.

This technique could be utilized to further evaluate disease entities that are nebulous for the bedside clinician. For example, there is no clear cut off for use of galactomannan BAL level in patients with probable invasive aspergillosis. Current clinical guidelines from the Food and Drug Administration recommend using a cut off greater than one based on review of multiple small studies, but biopsy specimens are needed for definitive diagnosis, which are not always possible in critically ill patients.^15^ Postmortem clinical correlation could assist with future diagnosis of suspected cases of aspergillosis by improving our understanding of the sensitivity and specificity of galactomannan BAL in the setting of confirmed pathologic disease.

Beyond microbiological studies, this novel procedure would further BAL evaluation in patients with interstitial lung disease. Interstitial lung disease is often correlated with surgical pathology. However, attempts have been made to correlate BAL cellular counts with subsets of ILD to allow a non‐invasive means to support diagnosis.^16^ One of the limitations for diagnosing interstitial lung disease is the heterogeneous nature of most ILDs. BAL can be diagnostic for many ILDs such as hypersensitivity pneumonitis; however, there is inherent limitation in BAL collection due to lack of reliability of targeting diseased lung segments versus normal lung segments. Utilizing this procedure for this patient cohort would allow for additional research into the field and enable further cellular and pathologic correlation. Developing this technique may help advance our understanding of how specific subsegments and lobules are sampled more precisely during a routine BAL, and eventually help clinicians target more specific diseased subdivisions of the lung to improve diagnostic yield.

Some investigations illustrate expected lung anatomy sampled by BAL relative to saline volume instilled although leave unexplained the relationship with active lung disease. Our technique provides precise procedural‐pathologic correlation. Radiologic evidence is not well integrated into several infectious disease studies that establish a relationship between quantitative culture results and BAL; our technique superimposes radiologic and pathologic evidence of disease. Immediate post‐mortem BAL and biopsy of lung tissue provides evidence of sensitivity and specificity of a BAL.^17^ However, that does not address a crucial question: Can a BAL procedure be optimized based on imaging findings? This is empirically demonstrated through our technique as a proceduralist may use their radiologic assessment, intra‐procedural decision making, and three‐dimensional histopathologic data to refine clinical BAL technique. This technique enables additional projects evaluating the effect of different amounts of injected volume, types of suction, positioning of the patient, and utilization of a mini‐BAL catheter instead of standard BAL.

## 5 CONCLUSION

Our study evaluates the relationship between bronchoalveolar volume and anatomic lung distribution based on BAL performed on various lobes and sub‐segments. The differences between upper and lower lobe lavages are explained by the anatomical variants. This procedure could be further expanded by changing patient factors (positioning, use of local vs. general anesthesia, etc.) and by evaluating clinical correlations between microbiological or other laboratory findings in comparison to pathologically confirmed disease.

## AUTHOR CONTRIBUTIONS

Alexander R. Gross designed and coordinated the study, collected and analyzed data, and wrote the manuscript. Temitope Kehinde conceptualized the study and collected and analyzed data. Lindsey Morais conceptualized the study and collected and analyzed data. Marshall Hutchison collected and analyzed data and wrote the manuscript. Joy Grise designed the study, contributed expertise in methods, and collected data. Nada Mohamed collected and analyzed data and wrote the manuscript. Varun Badami collected and analyzed data and wrote the manuscript. Haroon Ahmed collected and analyzed data and wrote the manuscript. Matthew Zdilla contributed expertise to methods and study design, facilitated IRB approval, and analyzed data. Jeffrey A. Vos contributed expertise to methods and study design and analyzed data. Rachel Leonard analyzed data, provided clinical expertise, and wrote the manuscript.

## CONFLICT OF INTEREST STATEMENT

None of the authors have any conflict of interest to disclose. This work is conducted with institutional review board‐waived status.

## ETHICS STATEMENT

This work received IRB‐exemption as research involving cadavers does not meet the regulatory definition of “human subject research.” Consent and participation in this study is regulated by the ethical standards of the West Virginia Human Gift Registry program.

## Supporting information


**Figure S1:** Injection method for explant lungs ‐ (A) Explanted fresh lungs. (B) Dissection of main stem bronchus to visualize intended order bronchus for injection (optional). (C) BAL cannula wedged, black arrowhead. (D) Wedged BAL cannula with Leur‐lok syringe loaded with gelatinized barium sulfate with blue ink.

**Figure S2:** Injection method for in‐situ injection ‐ (A) Thoracic cavity exposed via post‐mortem technique to facilitate incision of mainstem bronchus where cannula is inserted. (B) Cannula wedged in‐situ with empty Leur‐lok syringe.

## Data Availability

Study data is available upon written request to the corresponding author.
