# Supplementary figures and images for: A novel gelatinized barium sulfate injection method for assessment of bronchoalveolar lavage parameters
Source: Clin Respir J. 2024 Jan 11;18(1):e13721. doi: 10.1111/crj.13721 (PMC10784628; doi:10.1111/crj.13721)

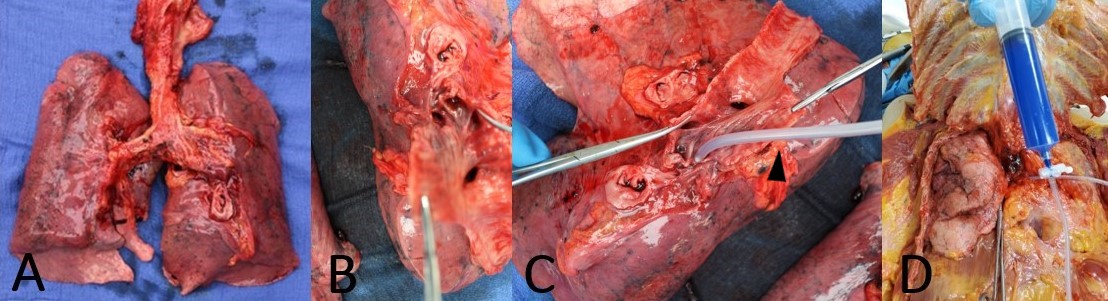

Supplement: Supplementary file 1 — Figure S1: Injection method for explant lungs ‐ (A) Explanted fresh lungs. (B) Dissection of main stem bronchus to visualize intended order bronchus for injection (optional). (C) BAL cannula wedged, black arrowhead. (D) Wedged BAL cannula with Leur‐lok syringe loaded with gelatinized barium sulfate with blue ink. [file CRJ-18-e13721-s001.jpg]

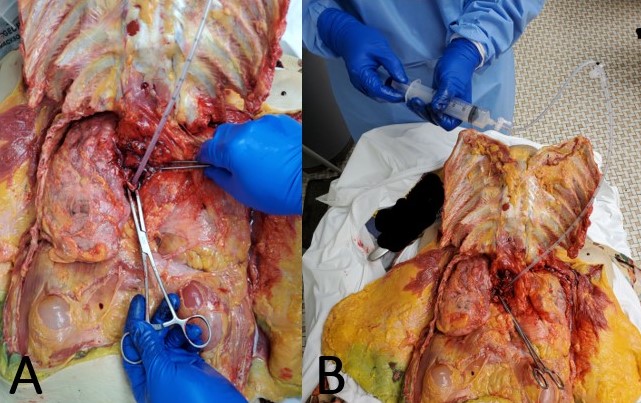

Supplement: Supplementary file 2 — Figure S2: Injection method for in‐situ injection ‐ (A) Thoracic cavity exposed via post‐mortem technique to facilitate incision of mainstem bronchus where cannula is inserted. (B) Cannula wedged in‐situ with empty Leur‐lok syringe. [file CRJ-18-e13721-s002.jpg]
